# A growing plastic smog, now estimated to be over 170 trillion plastic particles afloat in the world’s oceans—Urgent solutions required

**DOI:** 10.1371/journal.pone.0281596

**Published:** 2023-03-08

**Authors:** Marcus Eriksen, Win Cowger, Lisa M. Erdle, Scott Coffin, Patricia Villarrubia-Gómez, Charles J. Moore, Edward J. Carpenter, Robert H. Day, Martin Thiel, Chris Wilcox

**Affiliations:** 1 5 Gyres Institute, Los Angeles, California, United States of America; 2 University of California Riverside, Riverside, California, United States of America; 3 Moore Institute for Plastic Pollution Research, Long Beach, California, United States of America; 4 California State Water Resources Control Board, Sacramento, California, United States of America; 5 Stockholm Resilience Centre, Stockholm University, Stockholm, Sweden; 6 Algalita Marine Research and Education, Long Beach, California, United States of America; 7 EOS Center, San Francisco State University, Tiburon, California, United States of America; 8 ABR, Inc.--Environmental Research & Services, Fairbanks, Alaska, United States of America; 9 Facultad Ciencias del Mar, Universidad Católica del Norte (UCN), Coquimbo, Chile; 10 Center for Ecology and Sustainable Management of Oceanic Islands (ESMOI), Coquimbo, Chile; 11 Centro de Estudios Avanzados en Zonas Áridas (CEAZA), Coquimbo, Chile; 12 Minderoo Foundation, Perth, Western Australia, Australia; The University of Auckland - City Campus: University of Auckland, NEW ZEALAND

## Abstract

As global awareness, science, and policy interventions for plastic escalate, institutions around the world are seeking preventative strategies. Central to this is the need for precise global time series of plastic pollution with which we can assess whether implemented policies are effective, but at present we lack these data. To address this need, we used previously published and new data on floating ocean plastics (n = 11,777 stations) to create a global time-series that estimates the average counts and mass of small plastics in the ocean surface layer from 1979 to 2019. Today’s global abundance is estimated at approximately 82–358 trillion plastic particles weighing 1.1–4.9 million tonnes. We observed no clear detectable trend until 1990, a fluctuating but stagnant trend from then until 2005, and a rapid increase until the present. This observed acceleration of plastic densities in the world’s oceans, also reported for beaches around the globe, demands urgent international policy interventions.

## Introduction

Understanding the occurrence and trends of plastic abundance in the world are foundational to assessing current and potential future risks to humans and ecosystems [1]. Modeling plastic pollution’s fate and transport in the ocean surface layer (OSL) is complicated by complex mechanisms of degradation, fouling, and turbulent transport [2]. Fragmentation of large plastic results in micro- and nanoplastics leaving the OSL to shoreline and seafloor compartments, where they may cause harm to organisms through ingestion [3]. While recent modeling efforts suggest rapid export of plastic pollution away from the OSL [4], inputs are likely to continue [5, 6]. Therefore, understanding trends in regional and global plastic pollution mass and abundance is essential to evaluating and mitigating the risks.

While challenging, quantifying the global mass of plastics has previously been estimated for the OSL at 93,000 to 578,000 tonnes [7–9]. Spatial and temporal data gaps and variability in station-selection, sample-collection, and analysis make interpreting snapshots in time challenging and make establishing a trend even more difficult for the OSL [10]. Edelson et al. [11] suggested that the wide variability in reported inputs reveals an urgent need for improved monitoring frameworks to facilitate global governance.

A few earlier trends offer a perspective of plastic accumulation in the oceans. Archived Continuous Plankton Recorder (CPR) samples show an increasing trend of microfibers since the 1960s [12] and an increasing trend of macroplastic entanglement since the late 1950s [13]. Day and Shaw [14] reported an increase of microplastics in the North Pacific between 1976 and 1985, and Wilcox et al. [15] observed an increasing trend in the western North Atlantic from 1986 to 2015, with a rate of increase paralleling global cumulative plastic production. Although these studies suggest long-term increases, they are only from northern oceans surrounded by the most industrialized countries. In contrast, other studies have found no evidence of a rise in plastic pollution over time [see references in [10]].

Here, we evaluate a global dataset with all available historical data to provide an estimate of the temporal tendencies of plastic concentrations in the global OSL. We also offer a historic overview of international policy measures to reduce plastic inputs; based on that evaluation we call for urgent and effective solutions.

## Materials and methods

### Data sets: Net-tow sample collection and analysis

**Fig 1:**
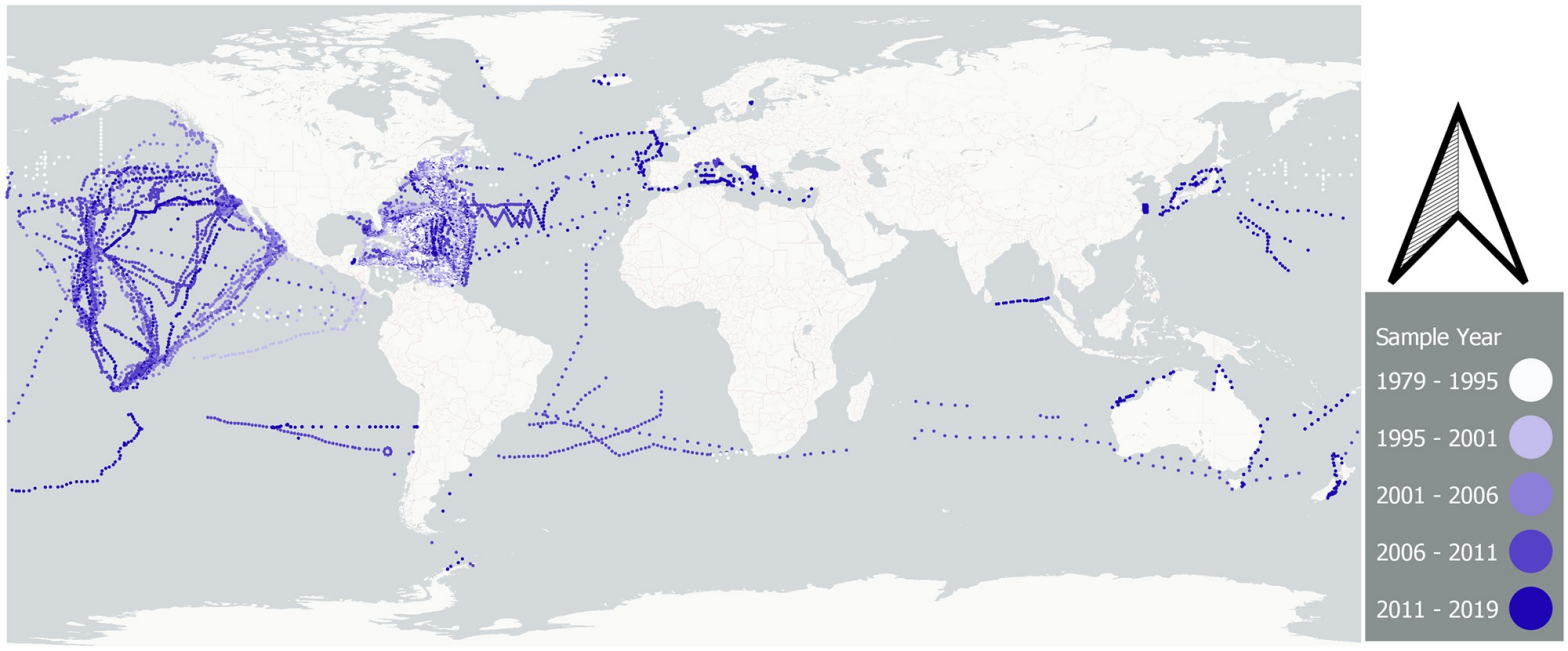
Map of sample station distribution. A total of 11,777 stations were used to model a global time trend.

We compiled data on OSL plastic abundance and distribution from published literature and unpublished sources, totaling 11,777 stations used in this trend analysis (Fig 1; S1 Dataset and on GitHub https://github.com/wincowgerDEV/ocean_plastic_modeling). Data were aggregated primarily from peer-reviewed manuscripts and previously unpublished data from 5 Gyres Institute expeditions. The data were collected with multiple methods of sea-surface sampling: manta trawl [8, 16], AVANI trawl [17], or a rectangular neuston net [7, 18]. We filtered the data to include samples with a lower mesh size range between 53μm and 505μm. Of the 11,777 samples in this study, 0.2% of samples were collected with a 53μm mesh (n = 27), 2.5% used 200μm (n = 303), 2.5% used 500–505μm (n = 293), and 94.7% used a 320–350μm (n = 11,154). The upper range of net openings was between 0.5m and 1m (S1 Table).

Although there was some variability in the methodology for each dataset, the methods typically were as follows: with the aid of a dissecting microscope, microplastics were manually separated from natural debris after being sorted through sieves [19, 20], then counted individually, before all microplastics from each size category were weighed together. To compute count data (in pieces km^-2^) we divided the total count of plastics collected by the surface area of water that the trawl went through. If this metric was not provided, we computed it using trawl dimensions and distance sampled as reported in the corresponding literature. If only the month and year were provided for the date, we used the 15th of the month specified as the day. Mass was estimated with a common conversion rate reported in the literature (1.36 x 10^−2^ g particle^-1^) by Morét-Ferguson et al. [21].

### Ocean surface-layer basins and currents

**Fig 2:**
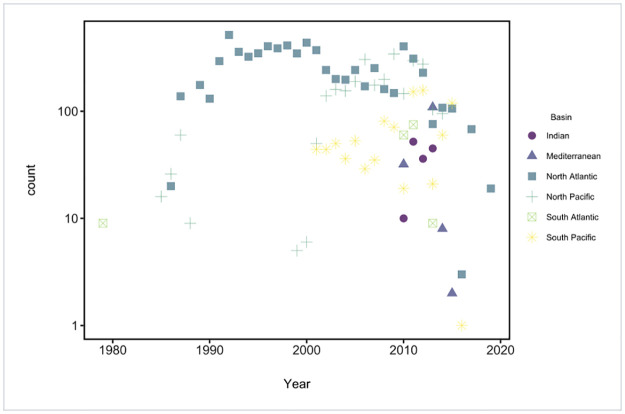
Count for each year for each basin. Some ocean basins are overrepresented (e.g., North Atlantic and North Pacific), whereas others have very few observations at a minority of time points (e.g., Indian, South Atlantic, South Pacific, and Mediterranean).

To improve estimates of OSL plastic abundance and distribution in different ocean basins, we assigned each station to one of six basins: North Atlantic, South Atlantic, North Pacific, South Pacific, Indian, and Mediterranean. The number of stations in each basin for each year is shown in Fig 2. We used model estimates for OSL plastic concentrations from Van Sebille et al. [9] and centered and scaled them within each ocean basin as a stable time reference to correct spatial sampling biases.

Wind and waves cause downward mixing of plastic particles at the OSL, decreasing the observed concentration when winds are strong [22]. This effect can be corrected by various process-based techniques when wind velocity is known [9, 15]. We corrected all data on ocean-surface concentrations by using the ERA-Interim Project [23] wind dataset, which provides average monthly surface wind speeds at (256 height x 512 width approximately 80 km) planet-wide spatial resolution and the Kukulka et al. [22] equation. We chose to use the Kukulka et al. [22] equation from 2012 instead of the refined Kukulka and Brunner [24] equation from 2015 because we incorporated previously published datasets that utilized the earlier 2012 equation [22], and we chose to be consistent in wind-corrected data. In cases where previous published data had been corrected, we acquired raw data and utilized the Kukulka et al. equation to standardize wind-corrected concentrations. The average expected effect of the wind correction is ~2.5 times the observed concentrations [22]. The expected ocean concentrations were derived by Lagrangian modeling of surface drifters for the year 2016 with a spatial resolution of 1° [9]. The concentrations in each basin were scaled and centered so that the expected concentration values would facilitate a regression analysis.

### Model

We modeled OSL temporal trends in plastic abundance and distribution with the above datasets corrected for wind and basin over time. Although there are many examples of global and regional plastic abundance and distribution estimates at a single point in time, temporal trends proved more challenging to produce.

Here, we used a model that removed the effects of site-bias and non-detects from the observed concentrations. We corrected for non-detects by treating zeros in the data as censored observations, in which the actual observation is below the detection level (1/volume of water sampled), instead of being considered a true zero value. We estimated the likely values of the zero records with the cenros function in the NADA package [25]; this function is a regression on ordered statistics that fits a model to the observed data and their quantiles to extrapolate the left-censored values. After correcting for non-detects with the cenros function, we estimated the actual values of the zeros to include in the model (S1 Fig). Plotting log-transformed observed concentration and expected concentration from an oceanographic transport model revealed an ocean-basin and concentration bias (quantile–quantile (qq) plot) (S2 Fig). To correct the collinear effect of expected concentration, we first fit a generalized additive model (GAM) model (gamma distribution) between log-transformed observed concentration and expected concentration from the van Sebille model [9]. We fit the model (concentration = expected concentration + basin) to the observed concentrations to get the residuals. Here the predicted density has no temporal component, only a spatial one, so the residuals from this relationship give a measure of how much an observation diverges from the relative concentration across space that we would expect. We then explored support for a time trend by modeling the residuals from the model above using a smooth function on time in a generalized additive model (S3 Fig). This approach is similar to adding all variables to a single model but allows us to interpret more precisely the effect of time by itself without influence from the other variables due to collinearity. We then estimated the average concentration change through time by multiplying the mean concentration observed by the reverse log-transformed residual fit. This approach allowed us to predict global plastic quantity weighted by observed mean plastic concentrations without influence from spatial effects.

Modeled results of floating microplastic item count (trillion of particles) and mass (millions of tonnes) globally for each year were calculated by averaging daily model results. Daily model results for particle counts were calculated by multiplying model results (particles km^-2^) x 361,900,000 km^2^ (ocean surface-area). Daily model results for mass were calculated by multiplying 1.36 x 10^−2^ g (average particle mass and weight from [21] x the number of particles.

## Results

**Fig 3:**
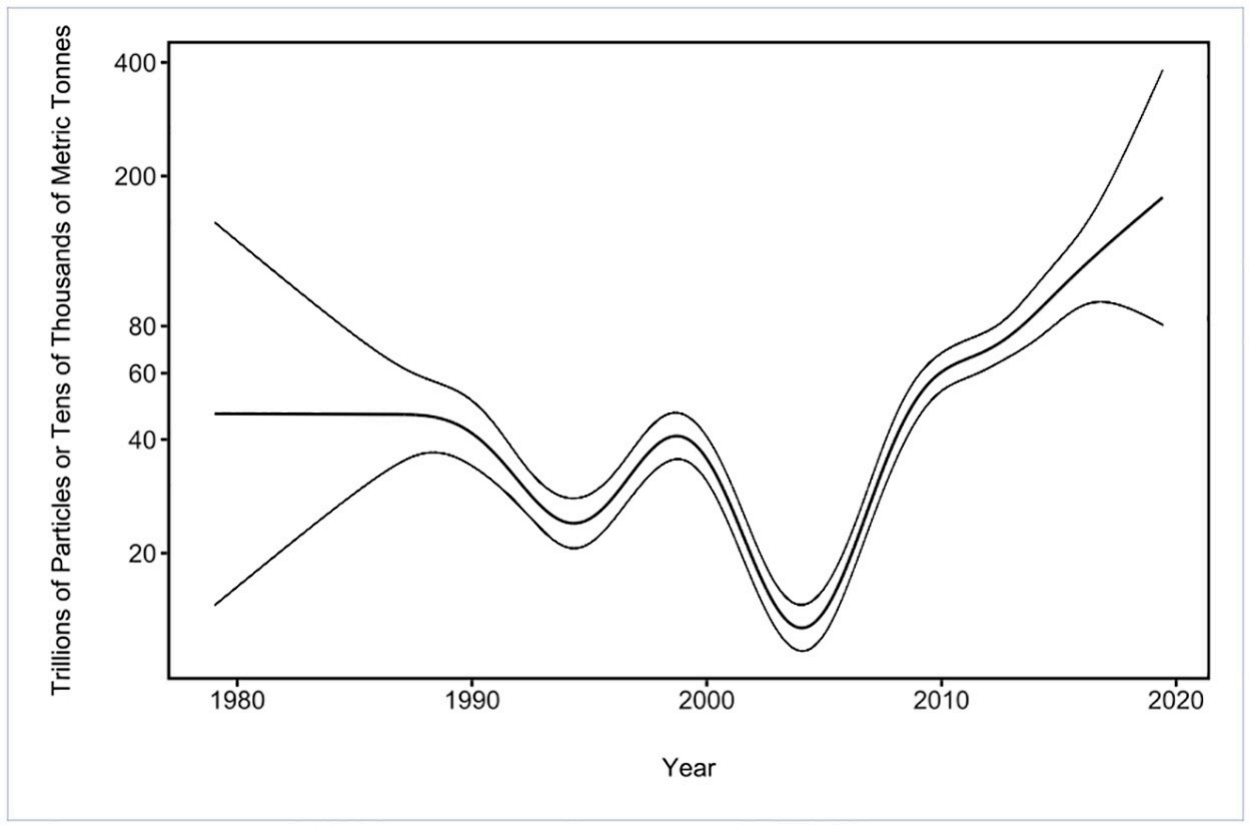
Global trend from 1979 to 2019 with confidence intervals. Change in the abundance of ocean plastic through time in trillions of particles or tens of thousands of metric tonnes using a smoothing spline and a generalized additive model. Central line is the model fit; confidence intervals are 2 times the standard error of the model.

After accounting for wind, site selection, and biases due to under-sampling, a significant trend was observed with ocean plastic through time (Fig 3). Plastic concentrations in the OSL varied over time, with a dramatic increase soon after the turn of the century. There are few samples before 1990, which is reflected in the width of the confidence intervals for the left-hand side of the temporal trend; however, during the last decade of the previous century the number of samples increased, resulting in reliable trends between 1990 and 2015 (Fig 3).

For the period with extensive sample coverage (1990–2015), there is substantial variability until 2004, which could be interpreted as stagnation or a decreasing trend; however, from 2005 onward, there is a consistent and rapid increase in plastic abundance (Fig 3). Based on our model results, we estimate that 82–358 trillion plastic particles (mean = 171 trillion plastic particles, primarily microplastics, weighing 1.1–4.9 million tonnes (mean = 2.3 million tonnes) were afloat in 2019 (Table 1).

**Table 1:** Estimated global mass and count of floating plastics from 1979 to 2019.

	Count (trillion particles)			Mass (million tonnes)			
Year	Mean	Lower estimate	Upper estimate	Mean	Lower estimate	Upper estimate	Number of samples
1979	46.82	15.40	142.50	0.64	0.21	1.94	9
1980	46.80	17.28	126.87	0.64	0.24	1.73	0
1981	46.77	19.42	112.77	0.64	0.26	1.53	0
1982	46.75	21.80	100.36	0.64	0.30	1.36	0
1983	46.73	24.43	89.47	0.64	0.33	1.22	0
1984	46.70	27.31	79.95	0.64	0.37	1.09	0
1985	46.68	30.38	71.80	0.63	0.41	0.98	16
1986	46.66	33.46	65.10	0.63	0.46	0.89	46
1987	46.53	36.02	60.13	0.63	0.49	0.82	198
1988	45.70	36.85	56.68	0.62	0.50	0.77	9
1989	43.37	35.39	53.15	0.59	0.48	0.72	176
1990	39.18	32.42	47.36	0.53	0.44	0.64	131
1991	33.76	28.75	39.66	0.46	0.39	0.54	294
1992	28.57	24.85	32.86	0.39	0.34	0.45	516
1993	25.09	21.70	29.00	0.34	0.30	0.39	359
1994	24.16	20.76	28.12	0.33	0.28	0.38	323
1995	26.11	22.61	30.17	0.36	0.31	0.41	348
1996	30.78	26.78	35.39	0.42	0.36	0.48	405
1997	36.71	31.78	42.41	0.50	0.43	0.58	387
1998	40.47	35.07	46.70	0.55	0.48	0.64	412
1999	38.57	33.64	44.21	0.52	0.46	0.60	352
2000	31.33	27.30	35.96	0.43	0.37	0.49	443
2001	22.75	19.77	26.18	0.31	0.27	0.36	466
2002	16.38	14.29	18.78	0.22	0.19	0.26	426
2003	13.23	11.53	15.18	0.18	0.16	0.21	410
2004	13.09	11.36	15.09	0.18	0.15	0.21	388
2005	16.05	14.02	18.38	0.22	0.19	0.25	486
2006	22.76	19.99	25.91	0.31	0.27	0.35	505
2007	33.41	29.18	38.26	0.45	0.40	0.52	463
2008	45.98	40.21	52.58	0.63	0.55	0.72	441
2009	56.57	50.12	63.84	0.77	0.68	0.87	561
2010	63.01	56.14	70.71	0.86	0.76	0.96	671
2011	67.17	59.86	75.36	0.91	0.81	1.02	886
2012	72.49	64.36	81.65	0.99	0.88	1.11	697
2013	81.02	70.02	93.76	1.10	0.95	1.28	364
2014	92.84	77.69	110.96	1.26	1.06	1.51	271
2015	106.89	86.74	131.75	1.45	1.18	1.79	227
2016	122.26	92.19	162.31	1.66	1.25	2.21	4
2017	138.86	91.18	211.94	1.89	1.24	2.88	68
2018	157.01	86.30	286.52	2.14	1.17	3.90	0
2019	171.16	81.98	357.56	2.33	1.11	4.86	19

Modeled results of floating microplastic item count (trillion particle count) and mass (million tonnes) globally for each year calculated by averaging daily model results. The lower and upper estimates are 2 times the standard error of the model. The number of sampling stations for each year also are presented.

Previous estimates of total ocean plastic concentrations distributed the model estimates back to ocean model grids [7–9]. The current estimate uses an oceanographic model to estimate concentrations by location, allowing us to extract a time trend without having to build a full spatio-temporal model. Then the model mean concentration prediction multiplied by the size of the ocean is used to predict the global ocean abundance; extrapolating the model predictions to ocean basins with few samples would have incorrectly estimated our certainty in the model grids.

## Discussion

Our study shows a significant increase since the turn of the century for the global ocean abundance and distribution of plastics in the OSL. The wide confidence intervals from 1979 to 1990 leave no clear detectable trend. From 1990 to 2005, concentrations of plastic fluctuate during this trendless period, followed by a dramatic increasing trend from 2006 onward. These observations may have been influenced by policy interventions, plastic production, fragmentation of existing floating plastic, and/or waste management and trade.

### Temporal trend

**Fig 4:**
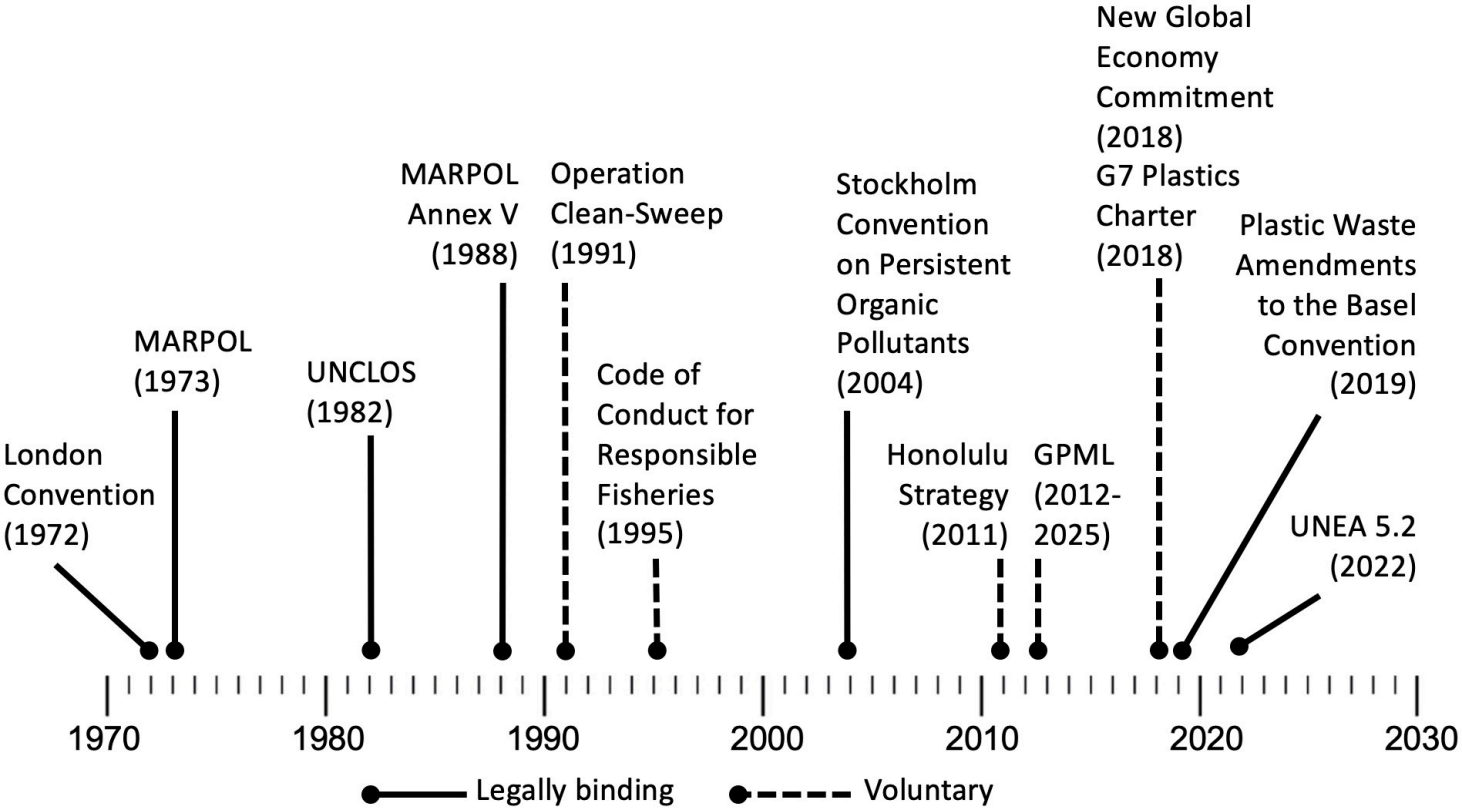
International policy interventions and maritime law. A cluster of binding international policy and maritime-law interventions preceding the millennium may have played a role in slowing the increasing trend of plastic waste in the OSL.

The stagnating trend observed prior to 2006 may have benefitted from important policy measures that were implemented during and just before that period (Fig 4). In 1988, MARPOL added Annex V, which established legally-binding agreements among 154 countries to end the discharge of plastics from naval, fishing, and shipping fleets. These interventions were preceded by the United Nations Convention on the Law of the Sea in 1982 and the Convention on the Prevention of Marine Plastic by Dumping of Wastes and other Matter in 1972 [26]. In 1991, the Plastic Industry Trade Association launched “Operation Clean Sweep” with a goal of zero loss of plastic pellets, powders, and flakes from factories [26], with decreasing pellet ingestion in biota observed [27]; however, these and others were voluntary agreements.

The rapid increase from 2005 onwards may reflect exponential growth of plastic production as it relates to inputs [15] or changes in terrestrial waste generation and management [28]. Older macroplastics adrift or stranded on shorelines or in rivers [4] continue to degrade and fragment and contribute to increases in the abundance of microplastics [15].

Despite the observed trends, there is uncertainty in collating data at a global scale. However, the dramatic increase observed herein for oceanic microplastics after 2005 parallels trends estimated for global beaches [29], which are based on completely independent observations. These parallel trends strongly suggest that plastic pollution in the world’s oceans during the past 15 years has reached unprecedented levels. Considerable data exist for the North Pacific and North Atlantic basins, although we lack information for the other ocean basins. More samples are needed in the South Atlantic, South Pacific, Mediterranean, and Indian oceans, all of which remain data-poor regions (see also [29]). In the future, if greater attention is given to the southern hemisphere, and sampling occurs at more-even intervals, models can provide outputs with higher resolution that can better identify spatial and temporal trends.

The globalization of raw plastic materials accelerated at the turn of the century [30] leading to rapid increases in import, export, and domestic production of plastic products and packaging and to increases in the amount of plastic waste generated. Simultaneously, plastic recycling, even in countries with highly developed waste-management infrastructure, has historically been low [6]. This lack of recycling has resulted in a flood of plastic products and packaging with dead-end material flows, largely due to the international trade of low-value waste plastics that remain as mismanaged waste in the receiving country [31]. The turn of the century also marked increased emissions of plastic waste from large fishing fleets and artisanal fisheries [32].

Increased international economic activity and the fragmentation and resuspension of degraded macroplastics probably drive the observed increasing trend. These factors have overwhelmed both the natural export mechanisms that transport plastic out of the OSL and any positive impact of those early binding interventions that may have driven the observed decrease toward the end of last century.

### Limitations

The study results are limited by sampling distribution and the unequal distribution of observations (Fig 2 and S4 Fig). The vast majority of samples was collected in the North Pacific and North Atlantic Oceans (Fig 2). Therefore, our results will be biased to observed trends in those ocean basins. The coefficient of variation (S4 Fig) had a wide spread (min = 0.447, max = 10.558, mean = 2.602), which underscores the challenge in modeling time-trends of ocean plastic concentrations accurately. The South Atlantic, Mediterranean, and Indian oceans had the lowest coefficient of variations, but that may be more due to the small number of samples and limited spatial distributions there than having smaller spatial variations.

In addition, we recognize limitations in our estimates of particle counts and mass. Particle counts may vary considerably when considering the lower size limit for particles. In terms of mass, and while we used a simple conversion of count to mass [12], new techniques are being developed (e.g., machine learning) to estimate mas and count conversions more accurately [33].

### Policy implications

The accelerating abundance of plastic in the OSL demands urgent international policy intervention to minimize ecological, social, and economic harm [5]. Without substantial widespread policy changes, the rate at which plastics enter aquatic environments will increase approximately 2.6-fold from 2016 to 2040 [6].

Existing international policies on plastic are fragmented, favor business-oriented solutions [34], lack specificity, and do not include measurable targets [35], with the exception of the 2019 Plastic Waste Amendments to the Basel Convention on the Control of Transboundary Movements of Hazardous Wastes and Their Disposal [36]. Although legally binding, this Convention regulates plastic trade only at the very end of its life cycle, once it becomes waste. International policy interventions after 2005 generally lack robust monitoring frameworks and enforcement mechanisms [34–37]. Because they also are non-binding and voluntary, they are not stemming the tide [38].

For example, the New Global Economy Commitment [39] promotes a set of circular economic principles to ensure the economic viability and meaningful reduction in the production of plastic waste: restrictions on certain products, extended producer responsibility for new and existing products, and waste-reduction targets. They invite stakeholders voluntarily to adopt the goal of making 100% of plastic packaging reusable, recyclable, or compostable by 2025, yet these measures advocate for private-sector self-regulation [34]. Economic barriers to recycled plastic continue to place virgin plastics at a lower cost, as the industries that produce and use plastics resist binding commitments to use recycled plastic and design standards for efficient recyclability, leaving recycling overall to fail expectations [40]. This increased production of virgin plastics will increasingly undermine circular economic principles, including the reuse economy and global policy interventions intended to reduce the most polluting plastic products and packaging [41].

## Conclusions

Although scientists continue to understand better the environmental fate of plastic pollution, there is consensus that global increases in plastic production result in dramatic increases in plastic pollution as shown herein, underscoring the urgent need for effective global governance. Currently, we are at a turning point in history. Early in 2022, at the United Nations Environmental Assembly 5.2. in Nairobi, all Member States adopted a resolution to end plastic pollution, committing to establish a legally binding global agreement that addresses the full life-cycle of plastic, including its production, design, and disposal, by 2024 [42]. The final outcome of this agreement will be a treaty, but its strength will depend on commitments by the member states and on whether measures are focused on the full life cycle of plastics, from extraction and manufacturing to its end of life [43]. Environmental recovery of plastic has limited merit, so solution strategies must address those systems that restrict emissions of plastic pollution in the first place. Therefore, establishing standardized monitoring frameworks to track global trends and creating binding and enforceable international agreements to prevent the emissions of plastic pollution are the best long-term global solutions.

## Supporting information

S1 Dataset These data are available at figshare.com. Plastic Marine Pollution Global Dataset, Modeling code, and Trend Reversal data are available open source On GitHub. The following dataset includes all data points used in our model: https://github.com/wincowgerDEV/ocean_plastic_modeling.
(TXT)

S1 Fig Correcting for non-detects. Correcting for non-detects using the cenros function, we estimated the actual values of the zeros to include in the model (A) observed concentration histogram log+1 transformed. Shows that there is a large gap between the zero observations and the nonzeros which indicates a need to correct non-detects. (B) corrected concentrations are normally distributed in log space.
(TIF)

S2 Fig QQ plots. (A) QQ plots for the initial model fit and (B) the residual model fit.
(TIF)

S3 Fig Global trend based on residuals. Annual global estimates of floating microplastic particles based on residuals from 1979 to 2019 show a period slow decreasing abundance and a steady increase from 2005 onward. Residuals are in log transformed space. The shaded grey area reflects confidence intervals. The “rugplot” at the bottom of the figure provides a line for each sample to show the density of stations. The low number of stations on either end of the figure results in wide confidence intervals.
(TIF)

S4 Fig Coefficient of variation for the datasets for each year. Sampling from year to year and basin to basin has a similar about of relative variability, which is a good thing in terms of fitting models to it.
(TIF)

S1 Table Summary of datasets used in model. The datasets used in the model (126 total). The year (of sample collection), data source, mesh size of net, the ocean basin, and the number of observations in that dataset are summarized.
(XLSX)
