# Supplementary figures and images for: A growing plastic smog, now estimated to be over 170 trillion plastic particles afloat in the world’s oceans—Urgent solutions required
Source: PLoS One. 2023 Mar 8;18(3):e0281596. doi: 10.1371/journal.pone.0281596 (PMC9994742; doi:10.1371/journal.pone.0281596)

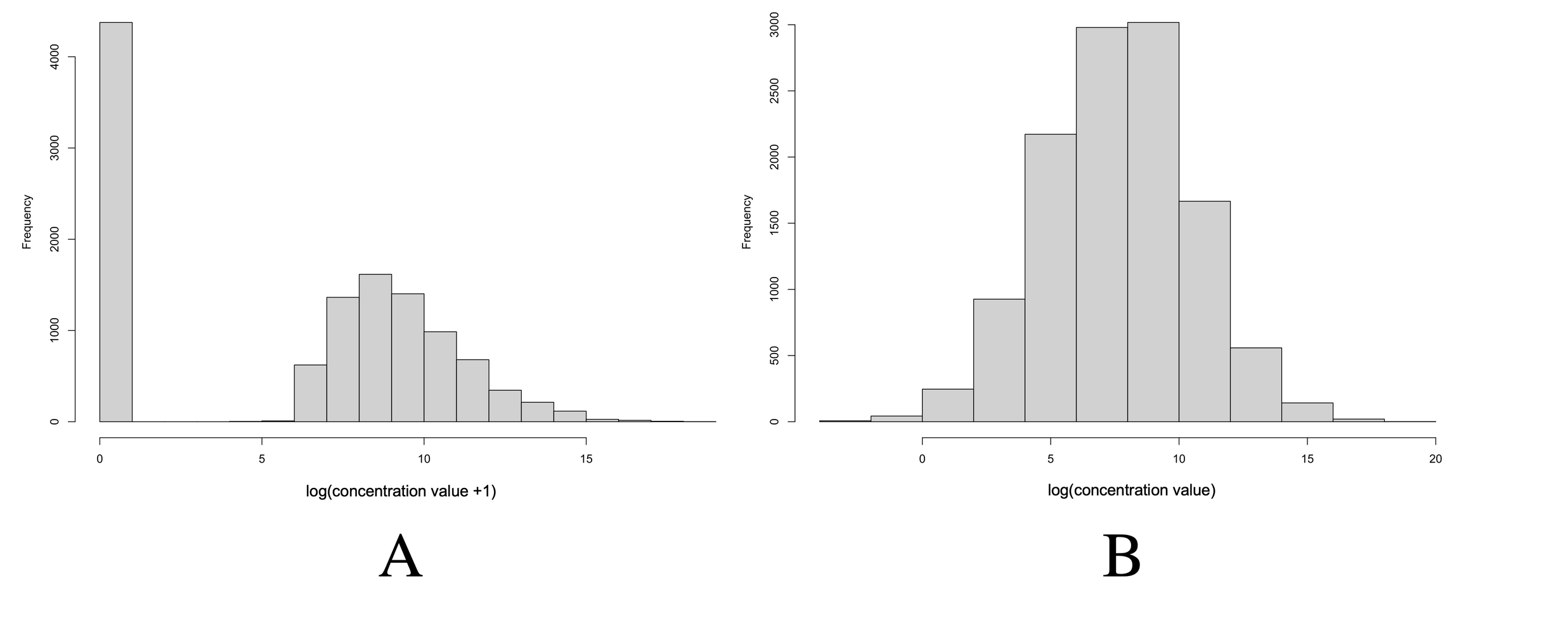

Supplement: S1 Fig — Correcting for non-detects using the cenros function, we estimated the actual values of the zeros to include in the model (A) observed concentration histogram log+1 transformed. Shows that there is a large gap between the zero observations and the nonzeros which indicates a need to correct non-detects. (B) corrected concentrations are normally distributed in log space. (TIF) [file pone.0281596.s002.tif]

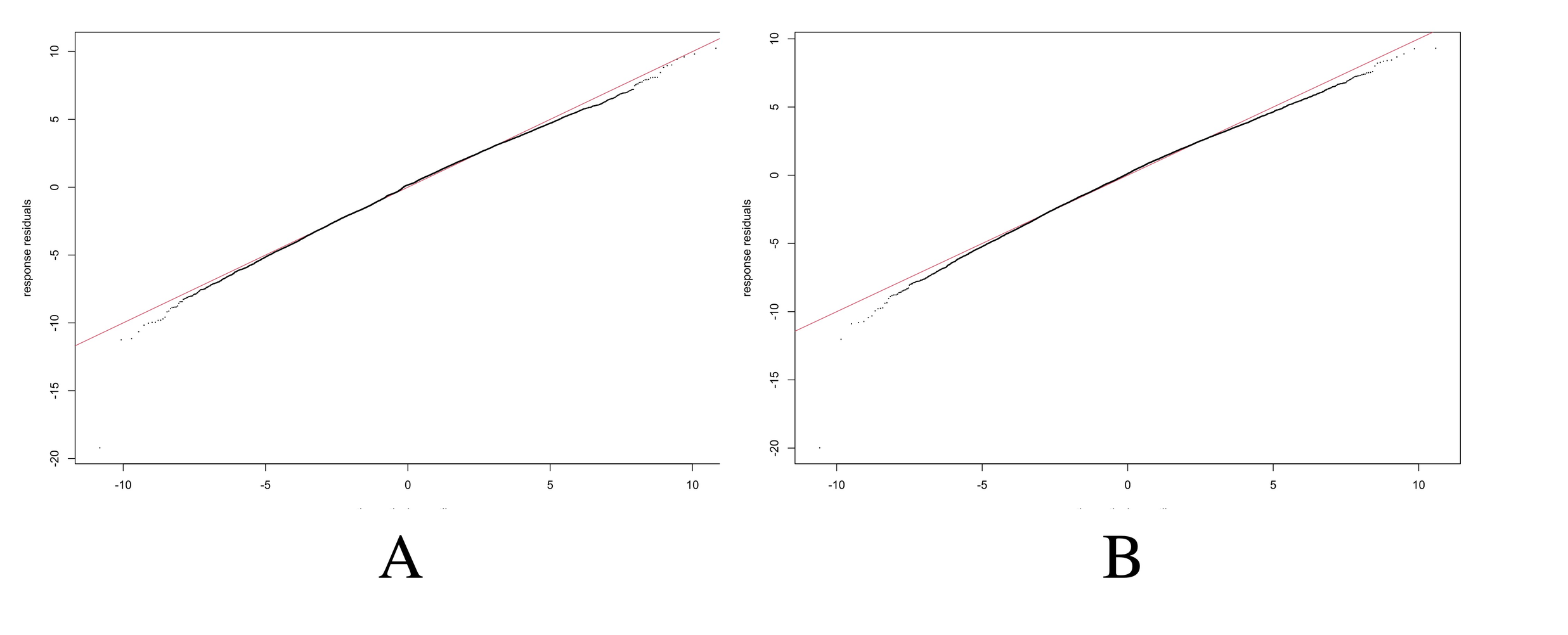

Supplement: S2 Fig — (A) QQ plots for the initial model fit and (B) the residual model fit. (TIF) [file pone.0281596.s003.tif]

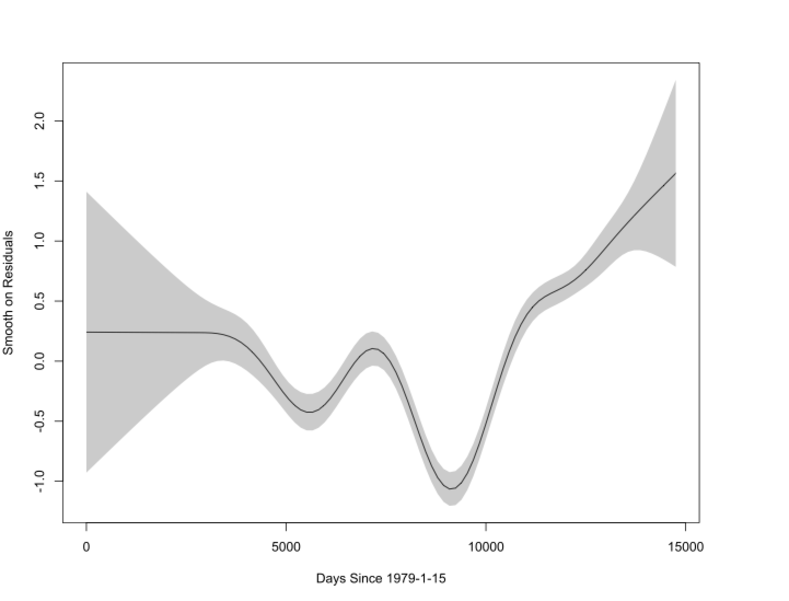

Supplement: S3 Fig — Annual global estimates of floating microplastic particles based on residuals from 1979 to 2019 show a period slow decreasing abundance and a steady increase from 2005 onward. Residuals are in log transformed space. The shaded grey area reflects confidence intervals. The “rugplot” at the bottom of the figure provides a line for each sample to show the density of stations. The low number of stations on either end of the figure results in wide confidence intervals. (TIF) [file pone.0281596.s004.tif]

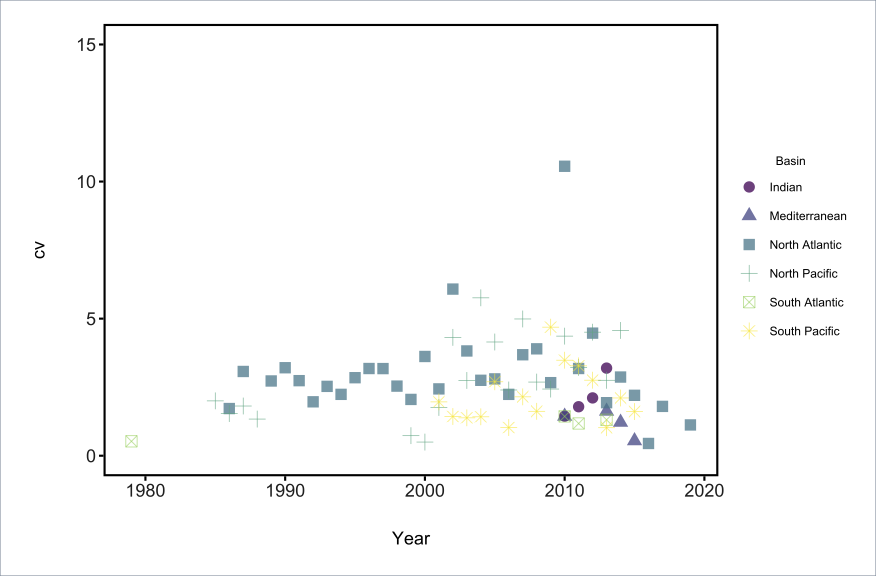

Supplement: S4 Fig — Sampling from year to year and basin to basin has a similar about of relative variability, which is a good thing in terms of fitting models to it. (TIF) [file pone.0281596.s005.tif]
